# From linezolid to contezolid: a strategy for safe continuation of therapy in complex tuberculosis

**DOI:** 10.3389/fmicb.2026.1852016

**Published:** 2026-07-17

**Authors:** Fei Lv, Rui Lin, Li Zheng, Mingming Zhang, Yanmei Liao, Guihui Wu

**Affiliations:** 1 Department of Pharmacy, Public Health Clinical Center of Chengdu, Chengdu, Sichuan, China; 2 Department of Tuberculosis, Public Health Clinical Center of Chengdu, Chengdu, Sichuan, China

**Keywords:** adverse events, contezolid, linezolid, real-world evidence, tuberculosis

## Abstract

**Background:**

Linezolid (Lzd) is a cornerstone for drug-resistant and complex tuberculosis (TB), but its utility is severely limited by hematological and neurological toxicities. Contezolid (Czd), a novel oxazolidinone, offers a potentially safer profile. However, real-world evidence remains limited.

**Objective:**

To evaluate the real-world effectiveness and safety of Czd in patients with complex TB, focusing on its role in managing Lzd toxicity through two distinct strategies: salvage therapy for Lzd-intolerant patients and proactive initiation in patients at high risk for Lzd-related adverse events.

**Methods:**

We conducted a retrospective study of TB patients treated with Czd-containing regimens. Patients were categorized into two groups: Group A switched from Lzd to Czd due to Lzd intolerance; Group B received Czd as initial treatment due to high risk of Lzd toxicity. Treatment outcomes, adverse events (AEs), and resolution of Lzd-related AEs after switching to Czd were analyzed.

**Results:**

All 31 patients in Group A experienced Lzd-related AEs (100%), with median onset of 45 days. Anemia was the most common AE (90%). After switching to Czd, significant recovery occurred in all AEs, with 52% achieving complete AE resolution. In Group B, no significant changes were observed. Treatment outcomes were excellent in both groups, with 100% clinical improvement and high sputum culture conversion rates (90% vs. 88%).

**Conclusion:**

Czd represented an effective salvage strategy for Lzd intolerance, significantly improving toxicity, ensuring therapeutic continuity, and suggesting preliminary evidence of maintained efficacy in complex TB. For patients at high risk of Lzd-related AEs, selecting Czd as initial therapy may prevent severe toxicity.

## Graphical abstract

**Figure:**
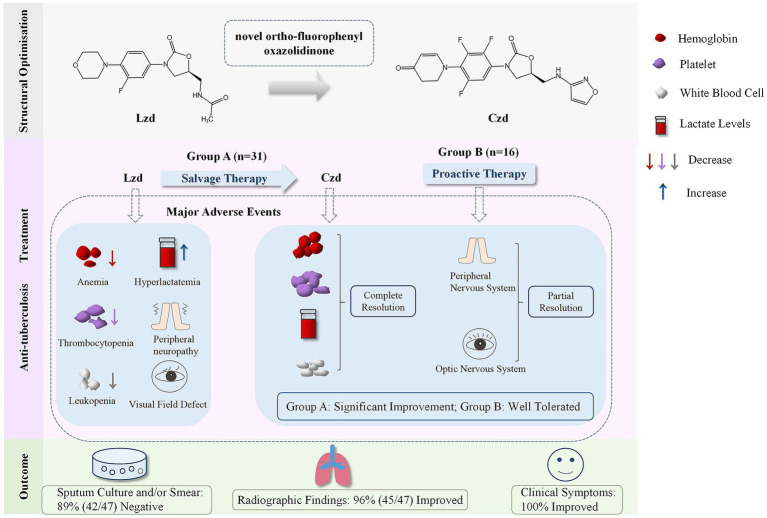
From linezolid to contezolid for safe continuation of therapy in complex tuberculosis.

## 1 Introduction

Tuberculosis (TB) remains a formidable global health challenge, with its persistence largely attributed to the emergence of complex treatment barriers. These challenges encompass prolonged treatment duration, severe adverse drug reactions, and increasing rates of drug resistance, which collectively contribute to suboptimal treatment adherence and clinical outcomes (Belete, 2022). The situation is particularly critical for multidrug-resistant TB (MDR-TB) and extensively drug-resistant TB (XDR-TB), where conventional treatment options are limited and often ineffective, leaving patients with few therapeutic alternatives. Amidst these therapeutic limitations, linezolid (Lzd) has emerged as an indispensable component of contemporary TB management. As a potent oxazolidinone antibiotic, Lzd has been formally recommended by the World Health Organization (WHO) for drug-resistant TB (DR-TB) treatment and serves as a cornerstone in modern regimens such as the Bedaquiline, Pretomanid, and Lzd (BPaL) regimen (Chen et al., 2026). Clinical evidence consistently demonstrates that Lzd-containing regimens significantly improve treatment outcomes in severe TB cases, including tuberculous meningitis, where it has been shown to enhance survival rates and accelerate clinical recovery (Fei et al., 2025). The broad implementation of Lzd across diverse clinical contexts, particularly its extensive use in China for managing MDR/XDR-TB cases, substantiates its fundamental therapeutic value (Zhang et al., 2022). This established clinical efficacy, coupled with widespread professional acceptance, firmly positions Lzd as an indispensable agent within the contemporary anti-TB pharmacopeia, especially for patients with severely constrained treatment alternatives.

Although Lzd holds an irreplaceable core position in the treatment of MDR/XDR-TB, its severe toxicities have become a major clinical challenge. The incidence of hematological toxicity reached up to 32.9%, while the peripheral neuropathy could reach 35.6% during the mid-to-late stages of treatment (Oktaviani et al., 2025). A recent study indicated that a daily dose ≥ 12 mg/kg based on body weight significantly increased the risk of peripheral neuropathy, anemia, and leukopenia. Therapeutic drug monitoring showed that a trough concentration (Cmin) > 2.0 mg/L is significantly associated with hematological toxicity, while the association between peripheral neuropathy and Cmin levels is more complex (Kuhlin et al., 2024). Notably, patients with comorbid diabetic peripheral neuropathy experienced a more pronounced decline in sensory nerve conduction velocity after Lzd treatment, indicating a higher risk of neurotoxicity in this population (Li et al., 2025). These adverse reactions led to premature treatment discontinuation in up to 40% of patients within three months (Kuhlin et al., 2024), significantly impacting treatment adherence and success rates. Therefore, effectively managing Lzd toxicity has become a critical clinical bottleneck, particularly for complex TB characterized by drug resistance, extensive disease (pulmonary and extrapulmonary), comorbidities, prior treatment failure, or intolerance to Lzd.

To overcome the clinical bottleneck posed by Lzd toxicity, Czd, a next-generation structurally optimized oxazolidinone antibiotic, showed significant promise. Its core advantage lies in replacing the morpholine ring present in Lzd with a 2,3-dihydropyridin-4-one (DHPO) ring, a unique structural modification that theoretically mitigates myelosuppression by reducing drug permeability into mitochondria (Wang and Ma, 2023). Existing evidence forms a complete chain from basic to clinical research: a preclinical study indicate that at equivalent doses (200 mg/kg/day), Lzd caused fatal myelosuppression in rats, while Czd is well-tolerated, with a no observed adverse effect level of 100 mg/kg/day (Wei et al., 2025). Clinical studies further confirm that Czd has anti-TB efficacy comparable to Lzd. A randomized controlled trial showed similar sputum culture conversion rates (92.9% vs. 92.3%) and lesion absorption rates (85.7% vs. 84.6%) after two months of treatment (Wang et al., 2025). Most importantly, multiple clinical studies consistently reported its significantly improved safety profile: during the 2-month treatment period, the AE incidence in the Czd group was only 14.3% (all gastrointestinal reactions), far lower than the 92.3% in the Lzd group, and no cases of myelosuppression or peripheral neuropathy were observed (Wang et al., 2023). In summary, current evidence demonstrates that Czd maintains efficacy comparable to Lzd while, due to its structural optimization, exhibits a significantly superior safety profile in early clinical data, particularly reflected in a substantial reduction of key toxicities, providing strong support for its role as an important clinical alternative.

Therefore, this study aims to evaluate Czd as an integrated strategy within a cohort of complex TB cases, including both Lzd-intolerant and high-risk patients. We systematically analyzed its efficacy in salvage therapy, toxicity reversal, and its value in proactively preventing toxicity in high-risk patients, thereby providing key clinical evidence for the use of Czd in TB treatment.

## 2 Materials and methods

### 2.1 Study design and patients

This was a single-center, retrospective, real-world study conducted at the Chengdu Public Health Clinical Center. All eligible cases were identified and enrolled through a search of the hospital information system. The collected data included demographic characteristics, tuberculosis history, detailed anti-tuberculosis treatment regimens (including drug composition, dosage, and duration), laboratory test results, adverse events, and treatment outcomes.

The inclusion criteria were as follows: (1) a confirmed diagnosis of tuberculosis (sputum culture positive), including both drug-susceptible and drug-resistant forms; (2) a treatment regimen containing Czd for a continuous period exceeding one month. Patients were excluded if they were lost to follow-up or had incomplete clinical records. A total of 47 patients met the criteria and were included in the final analysis. Based on their treatment pathways, they were categorized into two groups: Group A (Salvage Group, *n* = 31), comprising patients who switched to Czd due to intolerance to Lzd; and Group B (Proactive Group, *n* = 16), consisting of patients for whom Czd was selected as the initial treatment option based on the consideration of a high risk of Lzd toxicity. Specifically, among the 16 patients in Group B, one had pre-existing blurred vision and decreased visual acuity, one had severe thrombocytopenia, one had thrombocytopenia combined with elevated lactate, and the remaining 13 patients had concurrent decreases in all three hematopoietic lineages (platelets, hemoglobin, and white blood cells). These pre-existing conditions were considered contraindications or high-risk factors for Lzd use, leading to the proactive selection of Czd as the initial oxazolidinone agent.

This study was conducted in accordance with the ethical principles of the Declaration of Helsinki. The study protocol was reviewed and approved by the Ethics Committee of Chengdu Public Health Clinical Center (Approval No.: YJ-K2024-07-01). As this was a retrospective chart review that did not involve direct patient intervention, the requirement for obtaining informed consent was waived by the ethics committee. All patient data were anonymized prior to analysis.

### 2.2 Treatment regimens

All patients received individualized anti-TB regimens based on a comprehensive assessment of their clinical condition, treatment history, and available drug susceptibility profiles. Czd was administered orally at a dose of either 800 mg or 400 mg twice daily. Lzd, used prior to switching in Group A, was administered orally at a dose of either 600 mg or 300 mg once daily. The selected oxazolidinone was combined with a tailored background regimen of 3 to 4 other anti-TB drugs, which included options such as isoniazid (H), rifampicin (R), rifapentine (P), ethambutol (E), pyrazinamide (Z), moxifloxacin (Mfx), levofloxacin (Lfx), bedaquiline (Bdq), delamanid (Dlm), clofazimine (Cfz), cycloserine (Cs), and amikacin (Am). For patients in Group A, the core intervention involved switching from Lzd to Czd, with concomitant adjustments to the background regimen to maintain therapeutic efficacy. For patients in Group B, Czd was proactively selected as the initial oxazolidinone component to circumvent the risk of Lzd-associated toxicity.

### 2.3 Outcomes and assessments

Therapeutic outcomes were assessed based on sputum acid-fast bacillus (AFB) smear, *Mycobacterium tuberculosis* (MTB) culture conversion, clinical symptom resolution, and radiographic findings following treatment. Sputum culture conversion was assessed during the Czd containing treatment period. As this was a retrospective study, treatment duration varied among patients based on individual clinical conditions. However, each patient underwent at least one sputum culture assessment during the Czd treatment period. Safety evaluation was conducted through clinical manifestations of AEs, with severity graded according to the Common Terminology Criteria for Adverse Events (CTCAE, version 6.0) into Grade 1–2 (mild/moderate) and Grade 3–5 (severe/life-threatening/death). For patients in Group A who switched from Lzd to Czd, the degree of AE resolution following the switch was categorized as complete resolution (CR), significant resolution (SR), partial resolution (PR), or ongoing.

### 2.4 Statistical analyses

Statistical analyses were performed using IBM SPSS Statistics version 27.0 (IBM Corp., Armonk, NY, USA) and GraphPad Prism version 8.0 (GraphPad Software, San Diego, CA, USA). Continuous variables with non-normal distribution were expressed as medians with interquartile ranges (IQR) and compared between groups using the Mann–Whitney U test. Categorical variables were summarized as frequencies and percentages, and between-group comparisons were conducted using the Chi-square test or Fisher’s exact test, as appropriate. For within-group paired comparisons, the Wilcoxon signed-rank test was employed to evaluate changes in hematologic parameters (white blood cell count, platelet count and hemoglobin) and lactate levels at different time points. Kaplan–Meier survival analysis was performed to estimate the cumulative incidence of Lzd-related adverse events, and the median time to first adverse event was reported with its 95% confidence interval. Individual trajectory plots were generated using GraphPad Prism to visualize the dynamic changes in hematologic parameters and lactate levels over time. All statistical tests were two-sided, and a *p* value < 0.05 was considered statistically significant.

## 3 Results

### 3.1 Baseline characteristics and treatment outcomes

A total of 47 patients with complex tuberculosis who received Czd-containing regimens were enrolled, including 31 patients in Group A (salvage therapy) who switched from Lzd to Czd due to Lzd intolerance, and 16 patients in Group B (proactive therapy) who received Czd as initial treatment due to high risk of Lzd toxicity. In Group A, the age ranged from 18 to 78 years, while in Group B, the age ranged from 29 to 91 years. The baseline demographic and clinical characteristics of the two groups were generally comparable, with no significant differences observed in age, sex, resistance category, or type of tuberculosis (all *p* > 0.05, Table 1). Notably, Group B had numerically higher proportions of comorbidities associated with Lzd toxicity risk, including liver disease (44% vs. 26%), diabetes mellitus (25% vs. 13%), and HIV co-infection (13% vs. 3%), consistent with the clinical rationale for proactively selecting Czd in high-risk patients, although these differences did not reach statistical significance.

**Table 1:** Demographic and clinical data of the 47 patients who received Czd-containing anti-TB regimens.

Variable		Regimen of treatment		*p*-value
		Group A (Salvage group, *n* = 31)	Group B (Proactive group, *n* = 16)	
		Statistics	Statistics	
Median age (range)		55	57	0.425
Sex	Male	68% (21/31)	56% (9/16)	0.437
	Female	32% (10/31)	44% (7/16)	0.437
Resistance category	Drug-susceptible	55% (17/31)	81% (13/16)	0.074
	Rifampicin-resistant	13% (4/31)	13% (2/16)	1.000
	Multidrug-resistant	7% (2/31)	0%	0.541
	Extensively drug-resistant	18% (6/31)	6% (1/16)	0.396
	Other drug-resistant	7% (2/31)	0%	0.541
Patient treatment category	New	65% (20/31)	69% (11/16)	1.000
	Retreatment	35% (11/31)	31% (11/16)	1.000
Type of TB	Pulmonary TB only	16% (5/31)	6% (1/16)	0.648
	Extrapulmonary TB only	3% (1/31)	0%	1.000
	Pulmonary and extrapulmonary TB	81% (25/31)	94% (15/16)	0.396
Comorbidities	Liver disease	26% (8/31)	44% (7/16)	0.322
	Hypertension	23% (7/31)	6% (1/16)	0.234
	Diabetes mellitus	13% (4/31)	25% (4/16)	0.416
	Cancer	13% (4/31)	6% (1/16)	0.648
	Cardiovascular disease	7% (2/31)	0%	0.541
	Kidney failure	7% (2/31)	13% (2/16)	0.579
	HIV	3% (1/31)	13% (2/16)	0.264
	Psychiatric disorders	3% (1/31)	0%	1.000
	Systemic lupus erythematosus	0%	6% (1/16)	0.340
Use of background anti-TB drugs	Levofloxacin	58% (18/31)	88% (14/16)	0.04
	Isoniazid	61% (19/31)	75% (12/16)	0.347
	Ethambutol	61% (19/31)	69% (11/16)	0.614
	Cycloserine	65% (20/31)	31% (5/16)	0.03
	Pyrazinamide	55% (17/31)	19% (3/16)	0.018
	Rifampicin	35% (11/31)	19% (3/16)	<0.001
	Clofazimine	32% (10/31)	6% (1/16)	0.07
	Moxifloxacin	39% (12/31)	6% (1/16)	0.036
	Amikacin	29% (9/31)	13% (2/16)	0.287
	Prothionamide	19% (6/31)	13% (2/16)	0.697
	Bedaquiline	10% (3/31)	6% (1/16)	1.000
	Rifabutin	0%	6% (1/16)	0.340
	Delamanid	10% (3/31)	0%	0.541
	Para-aminosalicylic acid	6% (2/31)	0%	0.541
	Sitafloxacin	3% (1/31)	0%	1.000
	Rifapentine	3% (1/31)	0%	1.000
Treatment outcome	Clinical improvement	100%	100%	1.000
Sputum culture	Negative	90% (28/31)	88% (14/31)	1.000
	Positive	3% (1/31)	0%	1.000
	Unknown	7% (2/31)	12% (2/31)	0.597
Radiographic findings	Improved	94% (29/31)	100%	0.541
	Unchanged	6% (2/31)	0%	0.541

Regarding background anti-tuberculosis drug use, significant differences were observed between the two groups, reflecting their distinct therapeutic strategies. Group A had significantly higher usage of Cs (65% vs. 31%, *p* = 0.03), Z (55% vs. 19%, *p* = 0.018), R (35% vs. 19%, *p* < 0.001), and Mfx (39% vs. 6%, *p* = 0.036), while Group B had significantly higher usage of Lfx (88% vs. 58%, *p* = 0.04).

Treatment outcomes were excellent in both groups. All patients (100% in both groups) achieved clinical improvement. Sputum culture conversion rates were 90% in Group A and 88% in Group B (*p* = 1.000), and radiographic improvement was observed in 94% of patients in Group A and 100% in Group B (*p* = 0.541). Notably, the two patients (6%) in Group A with unchanged radiographic findings were both extensively drug-resistant (XDR-TB) cases, suggesting that severe drug resistance may limit radiographic response despite clinical improvement. These findings indicate that Czd-containing regimens were highly effective regardless of whether used as salvage therapy after Lzd failure or as initial treatment in high-risk patients.

### 3.2 Safety outcomes

**Figure 1:**
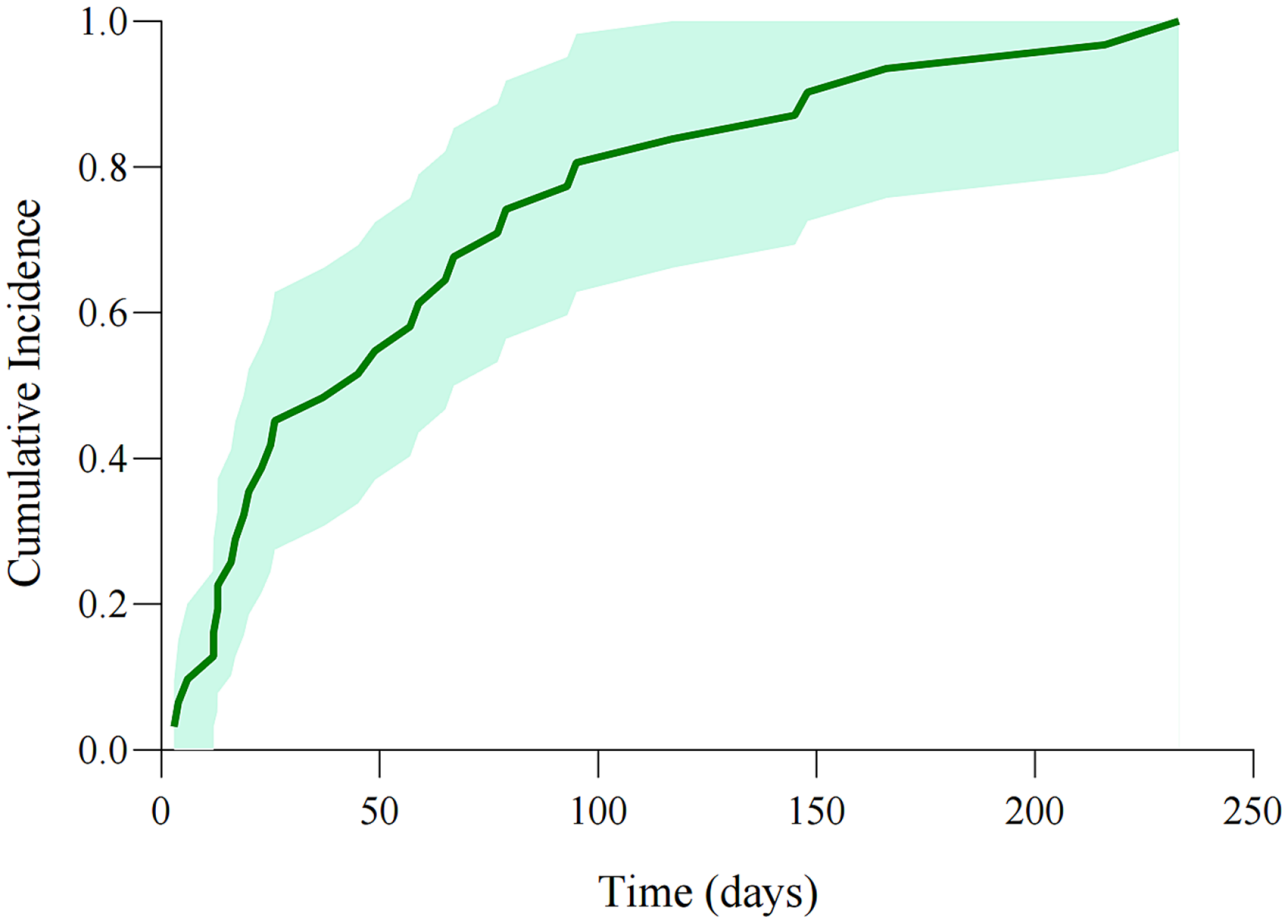
Cumulative incidence of Lzd-related AE (*N* = 31). Kaplan–Meier curve showing the time to first AE among patients treated with Lzd. The solid line represents the cumulative incidence, and the shaded area indicates the 95% CI calculated using the Greenwood formula. All 31 patients (100%) experienced at least one AE during follow-up. The median time to first AE was 45 days (95% CI: 10–80 days).

Kaplan–Meier analysis revealed that Lzd-related adverse events occurred early and universally in Group A. The cumulative incidence reached 38% by day 30, and all 31 patients (100%) experienced at least one adverse event during Lzd treatment (Figure 1). The median time to first adverse event was 45 days (95% CI: 10.1–79.9 days), with the majority of events occurring within the first 3 months of treatment (71%) (Table 2). Notably, 75% of patients discontinued Lzd within two months due to intolerable adverse events, underscoring the early and severe toxicity of Lzd in this population.

**Table 2:** Lzd-related AE and outcomes after switching to Czd in the Group A (*n* = 31).

Parameter	Proportion
Time from Lzd treatment to Lzd-related AE	
<3 months	71% (22/31)
≥3 and <6 months	22% (7/31)
≥6 months	7% (2/31)
Type of Lzd-related AE	
Anemia	90% (28/31)
Hyperlactatemia	61% (19/31)
Thrombocytopenia	42% (13/31)
Visual field defect	29% (9/31)
Leukopenia	29% (9/31)
Peripheral neuropathy	19% (6/31)
AE severity grade	
Grade 1	35% (11/31)
Grade 2	77% (24/31)
Grade 3	45% (14/31)
Grade 4	0%
AE outcome after switching to Czd	
Complete resolution	52% (16/31)
Significant resolution	7% (2/31)
Partial resolution	34% (11/31)
Ongoing	7% (2/31)

Complete resolution defined as resolution of all the signs/symptoms and recovery of abnormal laboratory test results; Significant resolution defined as resolution of most of the signs/symptoms and recovery of abnormal laboratory test results; Partial resolution defined as resolution of some of the signs/symptoms and recovery of some abnormal laboratory test results; Ongoing defined as persistence of most or all of the signs and symptoms and continued abnormality of laboratory test results without significant improvement.

Hematologic toxicities predominated, with anemia being the most common (90%), followed by hyperlactatemia (61%) and thrombocytopenia (42%). Visual field defect and leukopenia each occurred in 29% of patients, while peripheral neuropathy was observed in 19%. Severity assessment showed that most adverse events were grade 2 (77%) or grade 3 (45%), with only 35% being grade 1 and no grade 4 events reported.

After switching to Czd, the majority of patients showed substantial improvement in Lzd-related adverse events. Complete resolution was achieved in 52% of patients, significant resolution in 7%, and partial resolution in 34%. Only 7% of patients had ongoing adverse events at the time of data cutoff, which manifested as numbness of the limbs and decreased visual acuity. These findings demonstrate that while Lzd-related toxicity is almost universal in this complex patient population, switching to Czd effectively reverses the majority of these adverse events.

### 3.3 Impact of Czd on hematologic parameters and lactate levels in both groups

**Figure 2:**
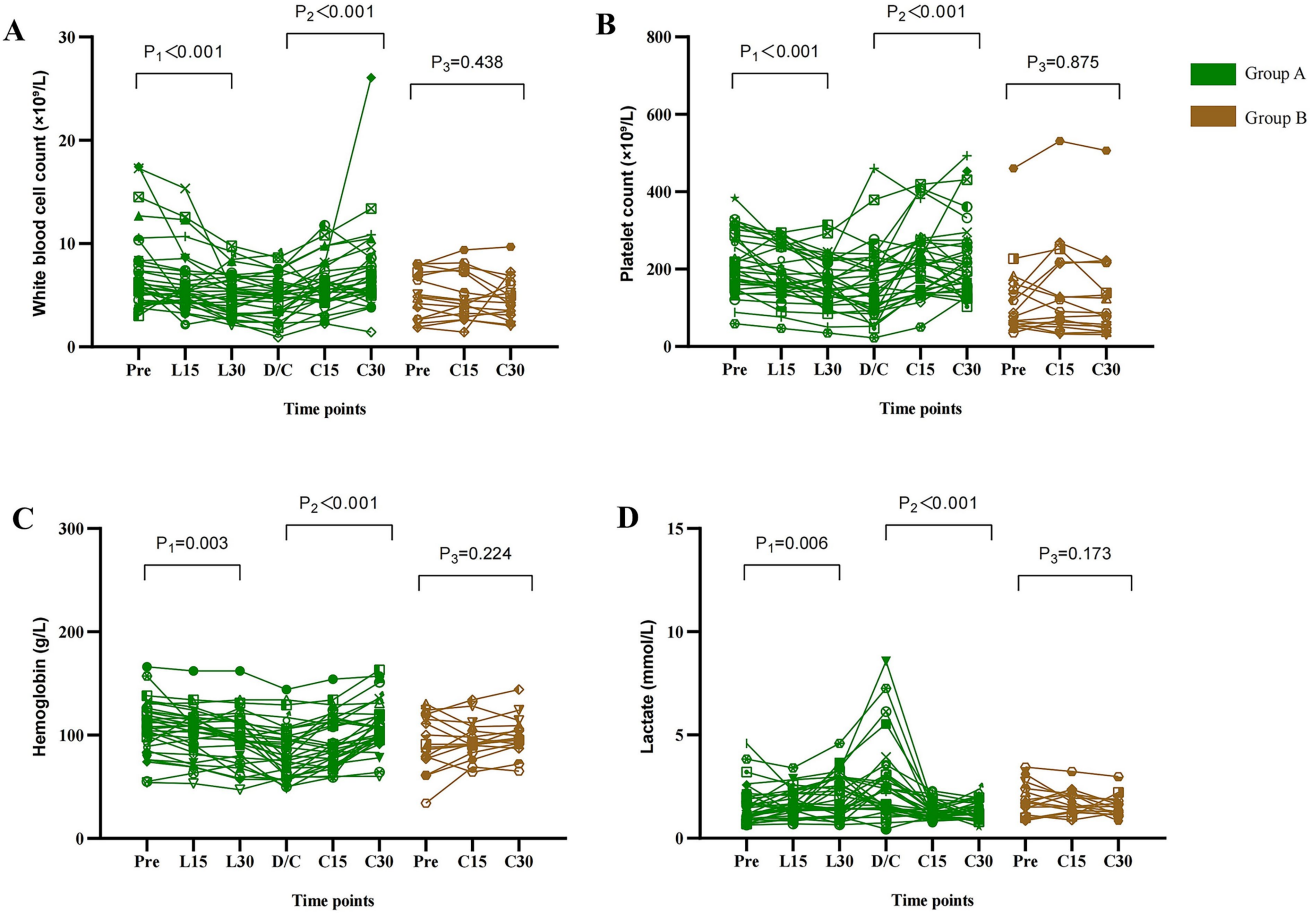
Individual trajectories of hematologic parameters and lactate in patients receiving Czd-containing regimens. Each panel shows individual patient trajectories for **(A)** white blood cell count, **(B)** platelet count, **(C)** hemoglobin, and **(D)** lactate levels. Each line in group A (salvage therapy, *n* = 31) represents an individual patient’s values at baseline (Pre), after 15 days (L15) and 30 days (L30) of Lzd treatment, at the time of Lzd discontinuation (D/C), and after 15 days (C15) and 30 days (C30) of Czd treatment. Each line in group B (proactive therapy, *n* = 16) represents values at baseline (Pre) and after 15 days (C15) and 30 days (C30) of Czd treatment (One patient in Group B was excluded from the lactate trajectory analysis due to missing blood lactate level data. No imputation was performed for missing values).

Figure 2 illustrates the individual trajectories of hematologic parameters and lactate levels. In Group A, significant deteriorations were observed during the first month of Lzd treatment. From baseline to day 30 of Lzd treatment, marked decreases were observed in white blood cell count (P1 < 0.001), platelet count (P1 < 0.001), and hemoglobin (P1 = 0.003), along with a significant increase in lactate levels (P1 = 0.006) (Figure 2).

After switching to Czd, all four parameters showed significant recovery by day 30 of Czd treatment compared to the time of Lzd discontinuation (P2 < 0.001 for all parameters). These results indicated that Czd effectively reversed Lzd-induced hematologic toxicities and lactate elevation, restoring these parameters toward baseline levels. In Group B, no significant changes were observed from baseline to day 30 of Czd treatment in any of the four parameters (all P3 > 0.05), demonstrating that Czd was well-tolerated with stable hematologic profiles throughout treatment when used as initial therapy (Table 3).

**Table 3:** Demographic and clinical details of 31 patients receiving contezolid-containing anti-TB regimens due to intolerance to linezolid treatment.

No.	Sex/Age	Diagnosis	Anti-TB regimen	Lzd	Lzd-Related AE and AE Grade	Time to AE	Czd	Outcome			Comment
								AE	Mtb	Symptoms	
1	M/29	Multi-organ TB	Bdq-Lzd-Cs-Am-Pas-Dlm; Pto-Czd-Cs-PAS	600 mg qd, 12 m	Leukopenia, Grade 2; Anemia, Grade 2; Peripheral neuropathy, Grade 2	6 m	400 mg bid, 12 m	PR	Negative	Improved	Received 2 m leukocyte elevation therapy after 5 m of Lzd; Dose interruptions of Lzd
2	M/78	PTB	Lzd-Lfx-Cs-Cfz-Pto for 5 m; Lzd-Cs-Cfz-E for 1 m; E-Cfz-Czd	600 mg qd, 6 m	Anemia, Grade 2; Peripheral neuropathy, Grade 2; Hyperlactatemia	3.5 m	400 mg bid, 10 m	CR	Negative	Improved	Received 0.5 m anemia correction therapy after 5.5 m of Lzd.
3	M/65	Multi-organ TB	Lfx-Lzd-Cs-Cfz-Z; Lfx-Czd-Cs-Cfz	600 mg qd, 1.5 m	Anemia, Grade 3; Thrombocytopenia Grade 2; Hyperlactatemia,	17 d	400 mg bid, 7 m	CR	Negative	Improved	Received 0.5 m anemia correction therapy after 1 m of Lzd; Dose interruptions of Lzd
4	M/72	Multi-organ TB	Lfx-Lzd-Cs-Cfz; CS-Czd-Lfx	300 mg qd, 4 m	Anemia, Grade 3; Hyperlactatemia; Thrombocytopenia, Grade 1	4 m	400 mg bid, 10 m	PR	Negative	Improved	Received 4 d anemia correction therapy during 2 m of Lzd; Dose interruptions of Lzd
5	M/25	Multi-organ TB	H-R-E-Z-Lfx-Lzd; H-R-E-Z-Lfx-Czd	600 mg qd, 1.5 m	Anemia, Grade 3	19 d	800 mg bid, 2 m	PR	Negative	Improved	Received 5d anemia correction therapy during 0.5 m of Lzd
6	F/40	Multi-organ TB	Lzd-Mfx-Cs-Cfz-Pto; Cs-E-Z-Am-Czd	600 mg qd, 1 m	Anemia, Grade 3	1 m	400 mg bid, 7 m	SR	Negative	Improved	Received 1d anemia correction therapy during 1 m of Lzd
7	M/53	Multi-organ TB	Z-L2-E-Lzd; Z-L2-E-Czd	300 mg qd, 4 m	Peripheral neuropathy, Grade 2; Anemia, Grade 3; Hyperlactatemia	3 d	800 mg bid, 2 m	ongoing	Negative	Improved	-
8	F/77	Multi-organ TB	H-R-Lzd-Mfx; H-R-Mfx-Czd	400 mg qd, 2 m	Visual field defect, Grade 2	15 d	400 mg bid, 3 m	CR	Negative	Improved	-
9	M/30	Multi-organ TB	H-R-E-Z-Mfx-Lzd; Cs-Z-Mfx-Czd	600 mg qd, 1 m	Anemia, Grade 3; Thrombocytopenia, Grade 2; Leukopenia, Grade 3	1 m	800 mg bid, 12 m	PR	Negative	Improved	Received 5 d anemia correction therapy during 0.5 m of Lzd.
10	M/20	Multi-organ TB	H-R-Z-E-Lzd; H-Lfx-Am-Czd	600 mg qd, 1 m	Leukopenia, Grade 4; Anemia, Grade 3; Thrombocytopenia, Grade 2; Hyperlactatemia	13 d	800 mg bid, 4 m	CR	Negative	Improved	Received 0.5 m therapy for anemia, leukocyte, and platelet correction during 0.5 m of Lzd
11	F/18	PTB	Bdq-Lzd-Lfx-Cfz-Cs; Czd-Bdq-Cfz-Lfx-Z	300 mg qd, 6 m	Anemia, Grade 1; Peripheral neuropathy, Grade 2	6 m	800 mg bid, 12 m	CR	Negative	Improved	-
12	M/69	Multi-organ TB	Lfx-Lzd-Cs; Cs-Czd-Lfx	600 mg qd, 300 mg qd, 2 m	Anemia, Grade 3; Leukopenia, Grade 1; Hyperlactatemia	1 m	400 mg bid, 9 m	CR	Negative	Improved	Received 4 d anemia correction therapy during 1 m of Lzd; Dose reductions of Lzd
13	M/61	Multi-organ TB	Lzd-Cs-Cfz-Pto; Mfx-Lzd-Cfz-Pto-Cs; Czd-Cfz-Cs-Pto-Z; Bdq-Dlm-Czd-Cfz-Cs	600 mg qd, 1 m	Anemia, Grade 2	1 m	400 mg bid, 6 m; 800 mg bid, 6 m	CR	Unknown	Improved	Received 3 d anemia correction therapy after 15 d Lzd
14	M/40	Multi-organ TB	H-Rb-E-Mfx-Lzd; H-Rb-Z-Mfx-Czd	600 mg qd, 2.5 m	Visual field defect, Grade 2; Anemia, Grade 2; Hyperlactatemia	2 m	800 mg bid, 1 m	PR	Negative	Improved	-
15	M/73	Multi-organ TB	Lzd-Lfx-Cs-Cfz-Z; Czd-Lfx-Cs-Cfz-Z	600 mg qd, 5 m	Hyperlactatemia; Anemia, Grade 3; Peripheral neuropathy, Grade 2; Visual field defect, Grade 2; Thrombocytopenia, Grade 1	5 m	800 mg bid, 3 m	CR	Negative	Improved	Received 5 d anemia correction therapy after 4 m of Lzd
16	M/73	Multi-organ TB	H-R-E-Z-Lzd; H-R-Z-E-Czd; H-Mfx-Czd	600 mg qd, 300 mg qd, 2 m	Anemia, Grade 3; Thrombocytopenia, Grade 3; Leukopenia, Grade 3	1 m	800 mg bid, 15d; 400 mg bid, 15 d	PR	Negative	Improved	Received 0.5 m therapy for anemia, platelet, and leukocyte correction after 1 m Lzd; Dose reductions of Lzd
17	M/71	PTB	H-R-Z-E-Lfx-Lzd; H-L2-E-Lfx-Czd	600 mg qd, 1 m	Anemia, Grade 3; Hyperlactatemia	1 m	800 mg bid, 1 m	Ongoing	Negative	Improved	Received 5 d anemia correction therapy during 0.5 m of Lzd
18	F/48	Multi-organ TB	Cs-Cfz-Pto-Lzd; Czd-Cs-Cfz-Am-Pto-PAS	600 mg qd, 1 m	Visual field defect, Grade 2	4 d	400 mg bid, 12 m	PR	Unknown	Improved	-
19	M/61	Multi-organ TB	H-Mfx-Lzd-Am; Cs-E-Mfx-Czd	600 mg qd, 1 m	Visual field defect, Grade 2; Anemia, Grade 3; Thrombocytopenia, Grade 3; Hyperlactatemia	6 d	800 mg bid, 3 m	PR	Negative	Improved	Received 15 d therapy for anemia and thrombocytopenia after 0.5 m of Lzd
20	M/62	PTB	Lfx-Cs-Lzd-Z; Lfx-Cs-Czd-Z	600 mg qd, 2 m	Visual field defect, Grade 2; Anemia, Grade 1; Hyperlactatemia	1.5 m	400 mg bid, 2.5 m	PR	Negative	Improved	-
21	F/75	Multi-organ TB	Lfx-Cs-Lzd-E-Am; H-Cfz-Mfx-Czd	600 mg qd, 3 m	Leukopenia, Grade 1; Anemia, Grade 2; Thrombocytopenia, Grade 1; Visual field defect, Grade 2; Hyperlactatemia	3 m	400 mg bid, 1 m	CR	Negative	Improved	Received 20 d therapy for anemia and leukocyte correction after 2 m of Lzd; Dose interruptions of Lzd
22	F/35	Multi-organ TB	H-R-E-Mfx-Lzd; H-E-Z-Mfx-Czd	600 mg qd, 1 m	Anemia, Grade 3; Hyperlactatemia; Leukopenia, Grade 1	1 m	800 mg bid, 3 m	CR	Negative	Improved	Received 10 d anemia correction therapy after 0.5 m Lzd
23	M/74	Multi-organ TB	H-R-E-Lfx-Lzd; H-R-E-Lfx-Czd	600 mg qd, 1 m	Anemia, Grade 3; Thrombocytopenia, Grade 2; Leukopenia, Grade 1; Hyperlactatemia	12 d	800 mg bid, 1 m	PR	Negative	Improved	Received 10 d therapy for anemia and platelet correction after 12 d Lzd.
24	F/39	Multi-organ TB	Pto-Mfx-Z-Lzd-Cs; Pto-Mfx-Z-Czd-Cs	600 mg qd, 3 m	Anemia, Grade 2; Thrombocytopenia, Grade 1; Hyperlactatemia	3 m	400 mg bid, 6 m	CR	Negative	Improved	Received 5 d anemia correction therapy after 1 m Lzd
25	M/52	Multi-organ TB	R-E-Lfx-Lzd; E-Lfx-Cs-Czd	600 mg qd, 2 m	Anemia, Grade 2; Thrombocytopenia, Grade 1	1 m	400 mg bid, 3 m	SR	Negative	Improved	Received 4 d anemia correction therapy after 1 m Lzd
26	F/27	Multi-organ TB	Lzd-H-Z-R; Am-Lfx-Czd	600 mg qd, 1 m	Anemia, Grade 2	13 d	800 mg bid, 1 m	CR	Negative	Improved	Received 15 d anemia correction therapy after 13 d Lzd
27	M/75	Multi-organ TB	H-E-Lfx-Lzd; H-E-Lfx-Czd	600 mg qd, 1 m	Anemia, Grade 2; Hyperlactatemia	12 d	400 mg bid, 1 m	PR	Positive	Improved	Received 10 d anemia correction therapy after 12 d Lzd
28	F/50	Lymph node TB	Bdq-Lzd-Mfx-Cs-Cfz; Czd-Z-Cs-Am	600 mg qd, 2 m	Anemia, Grade 2; Hyperlactatemia	1 m	400 mg bid, 8 m	CR	Negative	Improved	Received 13 d anemia correction therapy after 1 m Lzd
29	M/70	Multi-organ TB	H-R-Z-Mfx-Lzd; R-E-Z-Mfx-Cs-Czd	600 mg qd, 600 mg qod, 1 m	Anemia, Grade 2; Thrombocytopenia, Grade 2; Visual field defect, Grade 2	15 d	400 mg bid, 1 m	CR	Negative	Improved	Received 15 d therapy for anemia and platelet correction after 13 d Lzd; Dose reductions of Lzd
30	F/55	PTB	Mfx-Cs-Lzd-Cfz-Dlm; Mfx-Cs-Czd-Cfz-Dlm	600 mg qd, 6 m	Anemia, Grade 1; Thrombocytopenia, Grade 1; Leukopenia, Grade 1; Hyperlactatemia; Peripheral neuropathy, Grade 2	4 m	400 mg bid, 4 m	CR	Negative	Improved	-
31	M/55	Multi-organ TB	H-Mfx-Am-Lzd; Lzd-Cs-Cfz-Am	600 mg qd, 12 m	Visual field defect, Grade 2; Hyperlactatemia	3 m	400 mg bid, 8 m	CR	Negative	Improved	Dose interruptions of Lzd

TB, tuberculosis; PTB, pulmonary tuberculosis; M, male; F, female; H, isoniazid; R, rifampicin; Z, pyrazinamide; E, ethambutol; Czd, contezolid; Lzd, linezolid; Mfx, moxifloxacin; Cfz, clofazimine; Cs, cycloserine; Bdq, Bedaquiline; Dlm, Delamanid; Am, Amikacin; Lfx, levofloxacin; Pto, prothionamide; PAS, para-aminosalicylic acid; Rb, Rifabutin; CR, Complete resolution; PR, Partial resolution; SR, Significant resolution; AE, adverse event; Mtb, *Mycobacterium tuberculosis* detection by sputum culture and/or smear; m, month; d, day.

Collectively, these findings validate Czd as an effective salvage therapy that mitigates Lzd-induced adverse events while maintaining treatment continuity and support its proactive use in patients at high risk for Lzd intolerance.

## 4 Discussion

In this real-world retrospective study, we comprehensively evaluated the dual clinical strategy of Czd in complex tuberculosis patients: as salvage therapy after Lzd intolerance and as proactive initiation in patients at high risk for Lzd toxicity. The strongest evidence from our study concerns safety improvement. Our findings demonstrated that while Lzd-related AEs were nearly universal and occurred early, switching to Czd effectively reversed the majority of these AEs and restored hematologic parameters toward baseline levels. Furthermore, in patients who received Czd as initial therapy, no significant changes in hematologic parameters or lactate levels were observed, indicating a favorable safety profile. Thus, toxicity resolution and treatment continuity represent the primary contributions of this study.

The high burden of Lzd toxicity observed in our cohort aligns with previous reports. A systematic review by Oktaviani et al. (2025) reported that hematologic toxicity represents one of the most clinically significant adverse drug reactions associated with Lzd, with anemia affecting approximately one-third of patients. The incidence of hematological toxicity in our study was notably higher than previously reported, likely reflecting the complex nature of our patient population and the real-world setting. Notably, 80% of patients in Group A required active interventions during Lzd treatment, including granulocyte colony-stimulating factor for leukopenia, red blood cell transfusions for anemia, and oral medications such as iron polysaccharide complex, leucogen, and caffeic acid tablets to support hematologic recovery. Despite these supportive measures, the severity of Lzd-induced toxicity necessitated treatment discontinuation in all cases, with parameter levels at the time of discontinuation remaining significantly below baseline (P1 < 0.001 for white blood cell count, platelet count, and hemoglobin; P1 = 0.006 for lactate). Additionally, 8 of 31 patients (26%) experienced repeated dose interruptions or reductions due to intolerable adverse events before ultimately discontinuing Lzd, underscoring the clinical challenge of managing Lzd toxicity even with active intervention. These findings are consistent with the dose- and duration-dependent nature of Lzd toxicity described in the literature, where hematologic abnormalities typically emerge during the early months of therapy (Oktaviani et al., 2025).

Peripheral neuropathy was observed in 19% of our patients. This incidence was lower than in some cohorts, and the onset was later, consistent with the cumulative mitochondrial toxicity mechanism described by Xiong et al. (2024). In their study, Lzd induced peripheral neuropathy, with some cases failing to achieve complete resolution after switching to Czd. A similar pattern was observed in our study: at data cutoff, 7% of patients had persistent AEs characterized by limb numbness and decreased visual acuity.

The structural optimization of Czd replaces the morpholine ring with a 2,3-dihydropyridin-4-one (DHPO) ring. This modification has been shown to reduce mitochondrial permeability and mitigate myelosuppression (Chen et al., 2026). Our findings provide clinical evidence supporting this mechanistic advantage. After switching to Czd, significant recovery was observed in all hematologic parameters and lactate levels (P2 < 0.001 for all). Complete resolution of Lzd-related AEs was achieved in 52% of patients, while an additional 41% showed significant or partial improvement. Notably, the favorable safety profile of Czd was evident as only one patient (2%) in the entire cohort experienced a mild related AE (dry and itchy skin), which resolved with moisturizing.

In Group B, Czd was initiated proactively in patients at high risk for Lzd toxicity, including those with liver disease, diabetes, and HIV co-infection. No significant changes were observed in any hematologic parameters or lactate levels throughout treatment in this group (all P3 > 0.05). This stability, coupled with the absence of new AEs, supports the proactive use of Czd as a safer oxazolidinone option in populations at high risk for adverse reactions. These findings align with the accumulating evidence that Czd represents a promising alternative to Lzd, particularly for patients at risk of myelosuppression and neurotoxicity.

Despite the favorable preliminary findings regarding the tolerability and potential efficacy of Czd, economic considerations remain a significant barrier to its widespread adoption in China (Zhang et al., 2022). As of the data cutoff date (February 28, 2026), among the 47 patients receiving Czd, 18 had discontinued treatment. Of these discontinuations, 12 (67%) were attributed to financial burden, and the remaining 6 (33%) discontinued due to clinical decision-making or completion of their planned treatment course. No patient discontinued treatment due to Czd-related adverse events. Although Czd demonstrates superior tolerability compared to Lzd, its higher cost poses a substantial challenge for tuberculosis patients, who often face prolonged treatment duration and cumulative financial strain. Paradoxically, the repeated dose interruptions, extended treatment courses, and supportive interventions necessitated by Lzd toxicity also contribute to increased healthcare costs. Future pharmacoeconomic studies are warranted to comprehensively evaluate the cost-effectiveness of Czd versus Lzd in tuberculosis treatment. Such studies should consider both direct drug costs and indirect expenses associated with AE management and treatment prolongation.

The duration of Czd exposure in our cohort varied considerably. Among the 47 patients, the longest treatment duration exceeded 12 months, while the shortest was 1 month. Eighteen patients (38%) received Czd for more than 6 months. The remaining 29 patients were still under inpatient or outpatient follow-up. Given that 75% of patients in Group A discontinued Lzd within two months, we selected the 1-month time point for comparative analysis of hematologic parameters. Ongoing follow-up of these patients will provide valuable insights into the long-term safety and durability of Czd-containing regimens.

Several limitations of this study should be acknowledged. First, as a retrospective, single-center study with a relatively small sample size (*n* = 47), our findings may not be generalizable to broader populations. Second, the absence of randomization and the observational nature of the study may introduce selection bias, as reflected in the significant differences in background anti-tuberculosis drug use between groups. These differences were largely driven by individual patient characteristics, including disease severity, comorbidities, and baseline organ function (e.g., hematologic parameters, uric acid levels, liver and kidney function), which inherently influenced treatment decisions. Third, the relatively short follow-up period for some patients precludes assessment of long-term outcomes, including the durability of hematologic recovery and the potential for delayed AEs. Finally, the absence of pharmacoeconomic analysis limits our ability to provide guidance on the cost-effectiveness of Czd in resource-constrained settings. This issue is particularly relevant in China, where economic factors significantly influence treatment decisions.

Despite these limitations, this study provides valuable real-world evidence supporting the role of Czd as an effective salvage therapy for Lzd-intolerant patients and as a proactive strategy for high-risk individuals. The detailed characterization of Lzd toxicity, including the timing, severity, and types of adverse events, as well as the quantitative assessment of hematologic recovery after switching to Czd, offers clinically actionable insights. The inclusion of a proactive treatment group further strengthens the clinical relevance of our findings by demonstrating that Czd can be safely initiated in patients with baseline risk factors for Lzd toxicity.

## 5 Conclusion

In conclusion, our study demonstrates that Czd represents an effective salvage strategy for Lzd intolerance. It significantly improves toxicity outcomes and ensures therapeutic continuity, with preliminary evidence supporting maintained efficacy in complex TB. The stable hematologic profile observed in high-risk patients receiving Czd as initial therapy supports its proactive use to prevent Lzd-related toxicity. Future prospective, randomized controlled trials with larger sample sizes and longer follow-up are warranted to confirm these findings and establish optimal treatment strategies.

## Data Availability

The original contributions presented in the study are included in the article/supplementary material, further inquiries can be directed to the corresponding authors.
